# Metabolic engineering of bread wheat improves grain iron concentration and bioavailability

**DOI:** 10.1111/pbi.13074

**Published:** 2019-01-25

**Authors:** Jesse T. Beasley, Julien P. Bonneau, Jose T. Sánchez‐Palacios, Laura T. Moreno‐Moyano, Damien L. Callahan, Elad Tako, Raymond P. Glahn, Enzo Lombi, Alexander A. T. Johnson

**Affiliations:** ^1^ School of BioSciences The University of Melbourne Melbourne VIC Australia; ^2^ School of Life and Environmental Sciences Deakin University Burwood VIC Australia; ^3^ Robert W. Holley Center for Agriculture and Health USDA‐ARS Ithaca NY USA; ^4^ Future Industries Institute University of South Australia Mawson Lakes SA Australia; ^5^ Present address: Institute for Applied Ecology University of Canberra Canberra ACT 2617 Australia

**Keywords:** nicotianamine, 2′‐deoxymugineic acid, biofortification, Zinc, Caco‐2, X‐ray fluorescence microscopy

## Abstract

Bread wheat (*Triticum aestivum* L.) is cultivated on more land than any other crop and produces a fifth of the calories consumed by humans. Wheat endosperm is rich in starch yet contains low concentrations of dietary iron (Fe) and zinc (Zn). Biofortification is a micronutrient intervention aimed at increasing the density and bioavailability of essential vitamins and minerals in staple crops; Fe biofortification of wheat has proved challenging. In this study we employed constitutive expression (CE) of the rice (*Oryza sativa* L.) nicotianamine synthase 2 (*OsNAS2*) gene in bread wheat to up‐regulate biosynthesis of two low molecular weight metal chelators – nicotianamine (NA) and 2′‐deoxymugineic acid (DMA) – that play key roles in metal transport and nutrition. The CE‐*OsNAS2* plants accumulated higher concentrations of grain Fe, Zn, NA and DMA and synchrotron X‐ray fluorescence microscopy (XFM) revealed enhanced localization of Fe and Zn in endosperm and crease tissues, respectively. Iron bioavailability was increased in white flour milled from field‐grown CE‐*OsNAS2* grain and positively correlated with NA and DMA concentrations.

## Introduction

Micronutrient mineral deficiencies affect over two billion people worldwide with women and children most acutely at risk (Beal *et al*., 2017; Lopez *et al*., 2016). Iron (Fe) deficiency is the leading cause of anaemia, a condition that impairs cognitive development and work productivity and increases maternal and child mortality (Kassebaum *et al*., 2014; Lopez *et al*., 2016). Zinc (Zn) deficiency causes a variety of disorders including stunted growth in children. Human Fe and Zn deficiencies are most prevalent in SEA (Southeast Asia) and MENA (Middle East and North Africa), in part due to high consumption of micronutrient‐poor staple crops such as wheat (*Triticum aestivum* L.), rice (*Oryza sativa* L.) and maize (*Zea mays* L.) (Beal *et al*., 2017). Rising atmospheric CO_2_ concentrations will likely decrease Fe and Zn concentrations in C_3_ grains and could further exacerbate micronutrient deficiencies in SEA and MENA (Myers *et al*., 2014; Smith *et al*., 2017).

The wheat grain is comprised primarily of starch (60%–75%) and protein (12%–14%) with only low concentrations of Fe, Zn and other micronutrients (Shewry, 2009; Velu *et al*., 2014). Iron and Zn are remobilized from senescing vegetative tissues and/or translocated from roots during grain filling (Maillard *et al*., 2015; Pottier *et al*., 2014; Waters *et al*., 2009) and transported into developing grain via the vascular bundle in the crease region. In mature grain, the highest concentrations of Fe and Zn are found in the aleurone cells in conjunction with compounds such as phytic acid, polyphenols and fibre that inhibit bioavailability, while only low Fe and Zn concentrations are localized to endosperm tissues (Brouns *et al*., 2012; De Brier *et al*., 2016; Moore *et al*., 2012; Schlemmer *et al*., 2009; Singh *et al*., 2013; Stomph *et al*., 2011). Grain milling removes inhibitors of micronutrient bioavailability but also a significant proportion of the grain Fe and Zn (Hourston *et al*., 2017; Zhang *et al*., 2010).

Wheat biofortification aims to complement micronutrient supplementation and fortification programs by improving grain micronutrient density and/or bioavailability (Bouis *et al*., 2011; Prentice *et al*., 2017). Generation of Fe biofortified wheat through conventional breeding is hindered in modern wheat cultivars by inherently low grain Fe concentrations and a lack of genomic resources for this trait (Borrill *et al*., 2014; Velu *et al*., 2014), indicating that genetic engineering strategies may be required to generate novel genetic variation (Connorton *et al*., 2017; Singh *et al*., 2017).

Nicotianamine (NA) is a non‐protein amino acid formed by trimerization of S‐adenosylmethionine (SAM) in a process mediated by NA synthase (NAS) enzymes (Clemens *et al*., 2013). Nicotianamine plays key roles in the chelation and transport of metals such as Fe, Zn, manganese (Mn) and copper (Cu) in all higher plants (Takahashi *et al*., 2003; von Wiren *et al*., 1999). In graminaceous plant species, NA serves an additional purpose as the precursor to biosynthesis of 2′‐deoxymugenic acid (DMA), a root‐secreted phytosiderophore that chelates ferric Fe (Fe^3+^) in the rhizosphere and is subsequently re‐absorbed. Nicotianamine and/or DMA are also the main chelators of Fe in white wheat flour (Eagling *et al*., 2014a), and increased NA is known to increase Fe bioavailability in polished rice (Lee *et al*., 2012; Trijatmiko *et al*., 2016; Zheng *et al*., 2010). For these reasons, increased NA biosynthesis has emerged as a popular strategy for Fe biofortification of cereals (Johnson *et al*., 2011; Lee *et al*., 2012; Singh *et al*., 2017; Trijatmiko *et al*., 2016).

In this study of glasshouse‐ and field‐grown plants, we demonstrate that constitutive expression of the rice *OsNAS2* gene (CE‐*OsNAS2*) in bread wheat causes not only significant Fe and Zn enrichment of whole grain and flour fractions but also increased concentrations of NA and DMA that are positively correlated with Fe bioavailability in CE‐*OsNAS2* white flour. Interestingly, our results indicate that Fe bioavailability is higher in NA and DMA‐enriched white flour regardless of Fe concentration and that white flour NA and DMA concentrations are more critical than absolute Fe concentration in determining Fe bioavailability. These results provide unique insights into cereal Fe biofortification and highlight the importance of metabolic engineering strategies that enhance not only micronutrient density but also promoters of micronutrient bioavailability.

## Results

### Generation of independent transformation events with constitutive *OsNAS2* expression and selection of biofortified material for advanced nutritional analysis

**Figure 1:**
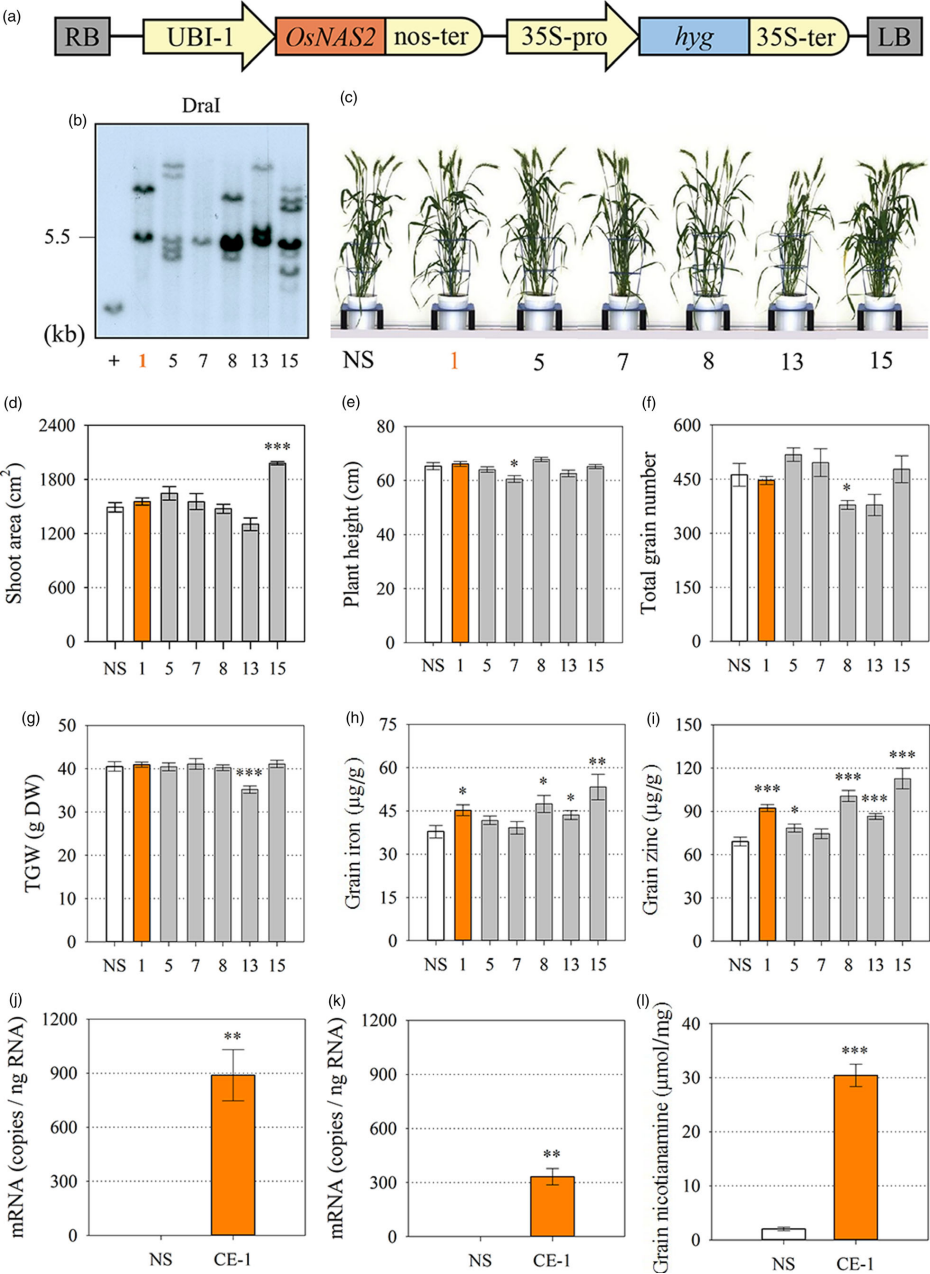
Generation and characterization of independent bread wheat transformation events constitutively expressing the rice nicotianamine synthase 2 (*OsNAS2*) gene. (a) Schematic representation of the T‐DNA construct. RB and LB: right and left borders, respectively; UBI‐1: maize ubiquitin 1 promoter; *OsNAS2*: rice nicotianamine synthase 2 gene (LOC_Os03 g19420); nos‐ter: nopaline synthase terminator; 35S‐pro: dual promoter of 35S cauliflower mosaic virus gene; *hyg*: hygromycin phosphotransferase gene; 35S‐ter: terminator of 35S cauliflower mosaic virus gene. (b) Southern blot analysis of T_1_ wheat events to determine T‐DNA insertion number. DraI: restriction endonuclease; + indicates positive control. (c) Representative plants of null segregant (NS) and the 6 transformation events (CE‐*OsNAS2*) 100 days after sowing. (d–g) Projected shoot area (cm^2^), plant height (cm), total grain number and thousand grain weight (TGW) of NS (white), leading CE‐*OsNAS2* event (CE‐1, orange) and other CE‐*OsNAS2* events (grey) at the T_1_ generation. Bars represent mean ± SEM of at least 7 biological replicates. (h, i) Iron and zinc concentration (μg/g DW) in T_2_ whole grain of NS, CE‐1 and other CE‐*OsNAS2* events. Bars represent mean ± SEM of at least seven biological replicates. (j, k) Relative quantification of *OsNAS2* transcript levels in NS and CE‐1 shoots and roots. Bars represent mean ± SEM of three bulked biological replicates, each with three technical replicates of quantitative RT‐PCR. (l) Nicotianamine concentration (μmol/mg) in whole grain of NS and CE‐1 plants at the T_2_ generation. Bars represent mean ± SEM of three biological replicates. Asterisks denote the significance between NS and CE‐*OsNAS2* events for *P* < 0.05 (*), *P* ≤ 0.01 (**), P ≤ 0.001 (***) as determined by student's *t*‐test. Wild‐type plants did not differ from NS plants for any trait measured and therefore only NS data is presented.

Bread wheat cultivar (cv.) Bobwhite transformants with constitutive expression (CE) of the rice nicotianamine synthase 2 (*OsNAS2*) gene were generated through biolistic transformation of a cassette containing the *OsNAS2* gene under regulatory control of the maize ubiquitin 1 promoter (UBI‐1) (Figure 1a). Six independent CE‐*OsNAS2* transformation events termed CE‐1, CE‐5, CE‐7, CE‐8, CE‐13 and CE‐15 were regenerated from tissue culture and Southern blot analysis showed that insert copy number ranged from 1 to 7 among the six events (Figure 1b). Automated imaging facilities at The Plant Accelerator (Adelaide, Australia) were used to phenotype T_1_ progeny of the six events; two events (CE‐1 and CE‐5) showed no phenotypic differences from a null segregant (NS) line derived from CE‐1 nor wild‐type (WT) wheat with respect to shoot area, plant height, total grain number and thousand grain weight (Figure 1c–g). Elemental analysis showed that four of the CE‐*OsNAS2* events (CE‐1, CE‐8, CE‐13, CE‐15) produced T_2_ grain with significantly increased Fe and Zn concentrations relative to NS and WT and one CE‐*OsNAS2* event (CE‐5) produced T_2_ grain with significantly increased Zn concentration relative to NS and WT (Figure 1h,i). Wild‐type plants did not differ from NS plants for any trait measured and had mean values of 1465 cm^2^ shoot area, 65 cm plant height; 375 total grain number, 38 g thousand grain weight, 40 μg/g DW grain Fe and 78 μg/g DW grain Zn.

Based on low insert copy number, no difference in plant phenotype, and increased grain Fe and Zn concentrations from the T_0_ to T_2_ generations, homozygous progeny of the double‐insert event CE‐1 and corresponding NS line were selected for a range of additional analyses from the T_3_ to T_6_ generation as depicted in Figure S1. Glasshouse‐grown CE‐1 seedlings displayed high *OsNAS2* expression in roots and shoots (Figure 1j,k). Expression of a range of endogenous *TaNAS*,* TaNAAT* and *TaDMAS* genes involved in NA and DMA biosynthesis was not significantly different between CE‐1 and NS seedlings, however, a trend towards slightly reduced expression was detected in CE‐1 (Figure S2). The CE‐1 and NS seedlings did not differ with respect to shoot Fe, Zn and DMA concentration while shoot NA concentration was 1.3‐fold higher in CE‐1 seedlings (Figure S3). Nicotianamine concentration was 15‐fold higher in CE‐1 mature grain relative to NS (Figure 1l).

### Constitutive *OsNAS2* expression alters Fe and Zn accumulation in grain, bracts, rachis and flag leaf at multiple points during grain filling

**Figure 2:**
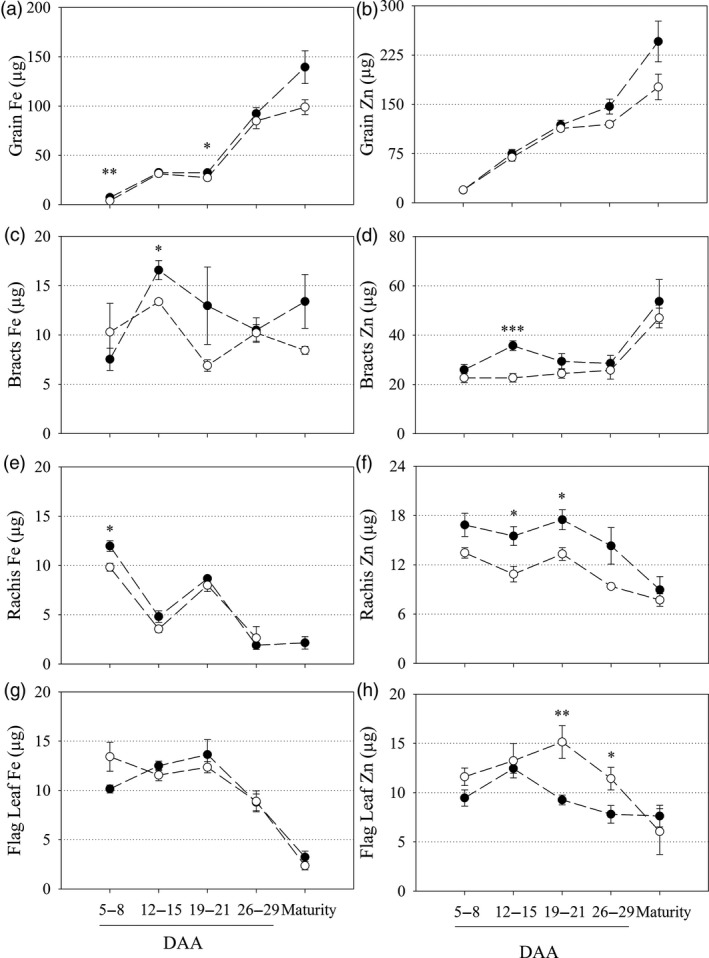
Fe and Zn content in vegetative and grain tissues during grain filling of CE‐*OsNAS2* and NS wheat lines. Fe and Zn content (μg) in NS (open circles) and CE‐1 (closed circles) plant tissues between 5–8, 12–15, 19–21 and 26–29 days after anthesis (DAA) as well as at maturity. (a, b) grain; (c, d) bracts; (e, f) rachis; and (g, h) flag leaf tissues were sampled for Fe and Zn content, respectively. Each symbol represents mean ± SEM of at least 3 biological replicates. Asterisks denote the significance between NS and CE‐1 for *P* < 0.05 (*), P ≤ 0.01 (**), *P* ≤ 0.001 (***) as determined by student's *t*‐test.

The main stem flag leaf, rachis, bracts and grain of glasshouse‐grown CE‐1 and NS plants were harvested at five timepoints post anthesis to determine Fe and Zn accumulation patterns in vegetative and grain tissues during grain filling. The CE‐1 grain had significantly higher Fe content at 5–8 days after anthesis (DAA) and 19–21 DAA relative to NS grain (*P* = 0.006 and *P* = 0.046; respectively) and showed non‐significant trends towards higher grain Fe and Zn content at maturity (Figure 2a,b). Bracts of CE‐1 plants had significantly higher Fe and Zn contents at 12–15 DAA relative to NS (*P* = 0.013 and *P* ≤ 0.001; respectively) but did not differ from NS bracts at maturity (Figure 2c,d). Rachis of CE‐1 plants had significantly higher Fe content at 5–8 DAA (*P* = 0.012), and significantly higher Zn contents at 12–15 DAA and 19–21 DAA (*P* = 0.014 and *P* = 0.020, respectively), but did not differ from NS rachis at maturity (Figure 2e,f). Flag leaf of CE‐1 and NS plants contained similar Fe content throughout grain filling. Conversely, CE‐1 flag leaf contained significantly lower Zn content at 19–21 DAA and 26–29 DAA relative to NS (*P* = 0.009 and *P* = 0.038, respectively) but did not differ from NS flag leaf at maturity (Figure 2g,h.).

Iron and Zn concentration data followed similar trends to the content data although more significant differences were detected (Table S1). Vegetative and grain tissue biomass was largely unchanged between CE‐1 and NS plants throughout anthesis and was not significantly different for any tissue at maturity (Table S2).

### Constitutive *OsNAS2* expression increases Fe accumulation in grain endosperm tissue and Fe and Zn accumulation within the crease region

**Figure 3:**
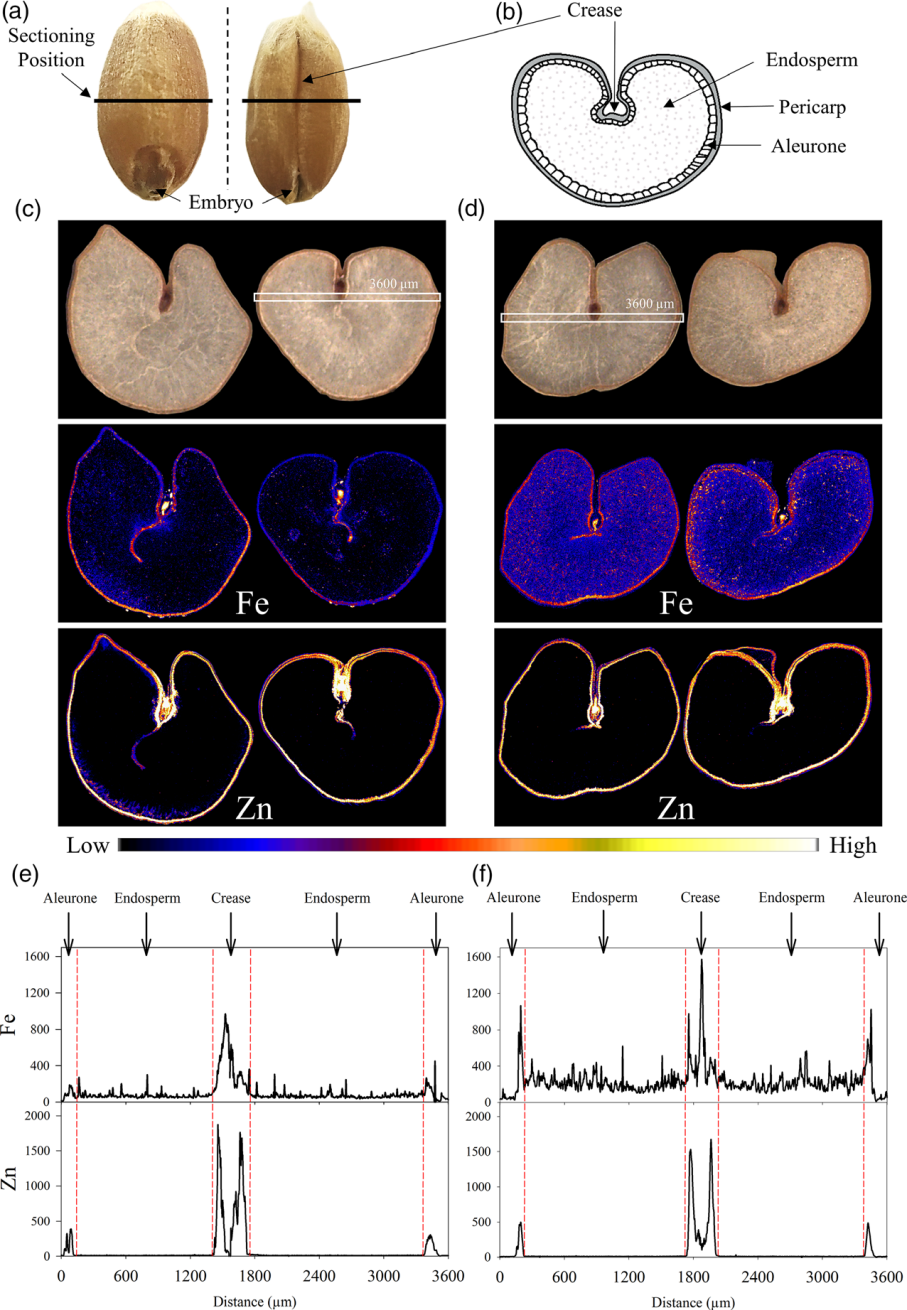
Distribution of Fe and Zn in CE‐*OsNAS2* and NS wheat grain. (a) Grain position where transverse cross‐sections were made. (b) Location of major tissue types in transverse section of wheat grain. (c) Bright field images of two representative NS grain sections and corresponding XFM elemental maps of Fe and Zn. (d) Bright field images of two representative CE‐1 grain sections and corresponding XFM elemental maps of Fe and Zn. Colour bar represents high (white) and low (black) elemental concentration. White boxes in the bright field images represent areas in two grain sections (one each for NS and CE‐1) used to generate line scans. (e) Line scans showing the distribution and signal intensity of Fe and Zn across NS grain. (f) Line scans showing the distribution and signal intensity of Fe and Zn across CE‐1 grain. Units for the *y*‐axis are elemental counts per pixel.

Elemental maps of Fe and Zn in transverse cross‐sections of two representative CE‐1 and NS grain showed that CE‐1 grain had higher Fe signal intensities in all grain tissue types relative to NS with the largest difference detected in endosperm tissues (Figure 3c,d). The Zn signal in both CE‐1 and NS grain was localized to the aleurone and crease regions and was not detectable in the endosperm. Line scans across the mid‐section demonstrated that CE‐1 grain had higher Fe signal intensities relative to NS in all regions of the grain, particularly in the endosperm, while CE‐1 grain had slightly higher Zn signal intensities in the aleurone cells relative to NS (Figure 3e,f). The line scans also revealed different Fe and Zn distribution patterns within the crease region of NS and CE‐1 grain, with Zn signals appearing as two distinct peaks on either side of the crease while Fe signals clustered into one central peak. Tri‐colour elemental maps of Fe, Zn and Cu distribution within the crease region demonstrated that these distinct peaks result from extensive Zn localization in modified aleurone cells bordering the crease in contrast to prominent Fe localization in the centrally located nucellar projection (Figure S4). While CE‐1 and NS grain showed similar Zn signal intensities in the modified aleurone cells of the crease, the CE‐1 grain had slightly higher Zn signal intensity in the nucellar projection relative to NS (Figure 3f). The CE‐1 and NS grain had similar distribution and signal intensities for Cu and Mn (Figure S5).

**Figure 4:**
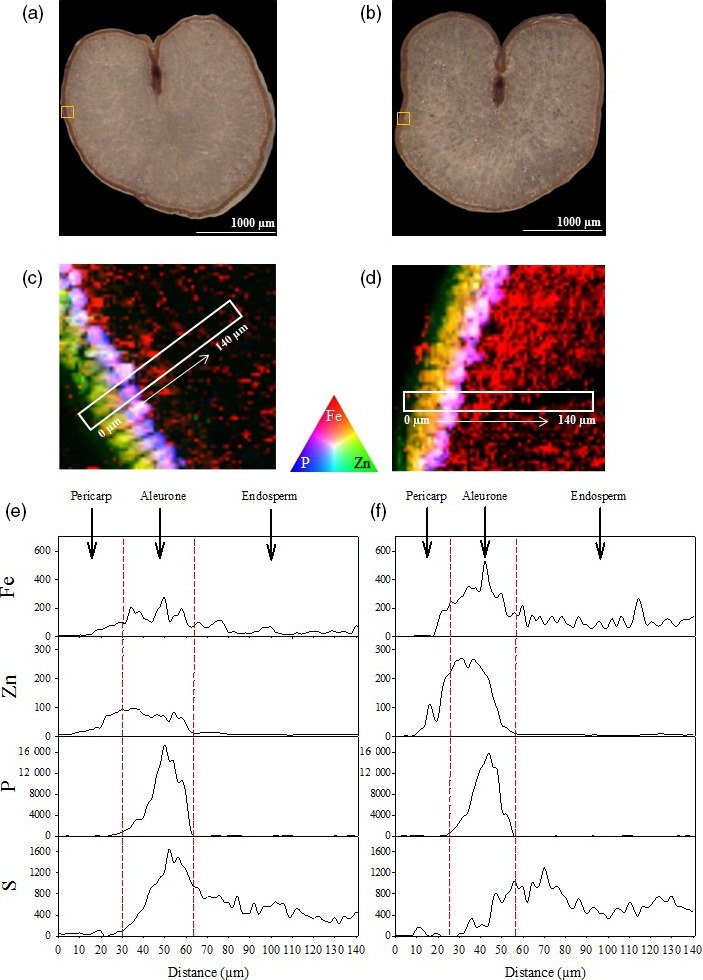
Distribution of Fe, Zn and P in CE‐*OsNAS2* and NS wheat grain. (a, b) Bright field images of NS and CE‐1 grain sections, respectively. Yellow boxes represent areas used to generate tri‐colour elemental maps. (c) Tri‐colour XFM elemental map of Fe (red), Zn (green) and P (blue) in NS grain. White box represents the area used to generate line scans. (d) Tri‐colour XFM elemental map of Fe (red), Zn (green) and P (blue) in CE‐1 grain. White box represents the area used to generate line scans. (e) Line scans showing the distribution and signal intensity of Fe, Zn, P and S in NS grain. (f) Line scans showing the distribution and signal intensity of Fe, Zn, P and S in CE‐1 grain. Units for the *y*‐axis are elemental counts per pixel.

Tri‐colour elemental maps of Fe, Zn and phosphorus (P) near the grain edge of one representative CE‐1 and NS grain demonstrated that the Fe signal in CE‐1 grain was enhanced within pericarp, aleurone and endosperm regions relative to NS grain (Figure 4c,d). The Zn signal in CE‐1 grain was enhanced primarily within the pericarp and aleurone cells, relative to the NS, and co‐localized with Fe in that region. The P signal in both NS and CE‐1 grain did not differ in intensity nor distribution and was localized to the aleurone cells. The trends observed in CE‐1 and NS tri‐colour maps were further confirmed by line scans (Figure 4e,f). In both CE‐1 and NS grain, P was localized exclusively to the aleurone cells with equal signal intensity, indicating that the significant enrichment of Fe in CE‐1 endosperm was not associated with phytic acid (a P containing compound). Sulphur (S) distribution and intensity (a proxy for protein) appeared slightly reduced in the CE‐1 aleurone, relative to NS, but similar to NS in the endosperm (Figure 4e,f).

### Constitutive *OsNAS2* expression does not alter the phenotype of field‐grown plants and increases Fe, Zn, NA and DMA concentrations in grain and white flour

**Figure 5:**
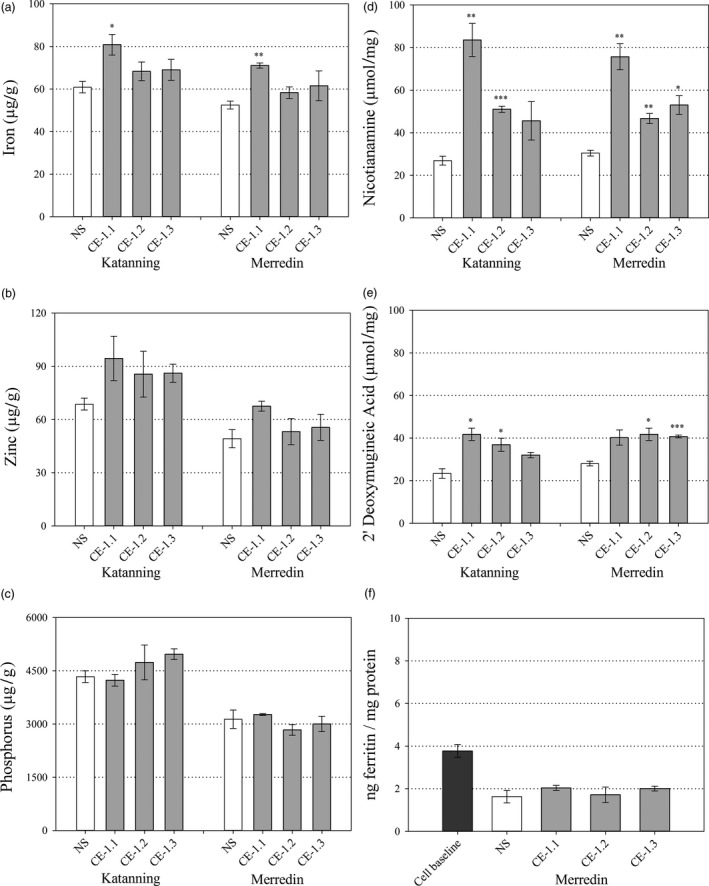
Whole grain nutrition of field grown CE‐1 and NS wheat lines. Nutrient and metabolite concentrations in whole grain samples of NS (white) and three CE‐1 sibling lines (CE‐1.1, 1.2 and 1.3, grey) at the T_6_ generation. (a–c) Whole grain Fe, Zn and P concentrations (μg/g) of NS and CE‐1 plants grown at Katanning and Merredin field sites. (d, e) Whole grain NA and DMA concentrations (μmol/mg) of NS and CE‐1 plants grown at Katanning and Merredin field sites. (f) Whole grain Fe bioavailability of NS and CE‐1 plants grown at Merredin field site. Bars represent mean ± SEM of 3 biological replicates. Asterisks denote the significance between NS and each CE‐1 wheat line for *P* < 0.05 (*), *P* ≤ 0.01 (**), *P* ≤ 0.001 (***) as determined by student's *t*‐test.

Replicated plots of three CE‐1 sibling lines designated CE‐1.1, CE‐1.2 and CE‐1.3 and the NS were evaluated in confined field trials at Katanning and Merredin, Western Australia in 2015. With the exception of CE‐1.1 at Merredin, phenotype of the CE‐1 sibling lines and NS did not differ with respect to plant height, spike number, biomass, thousand grain weight (TGW) and grain yield (Table S3). Whole grain Fe concentrations were significantly higher for CE‐1.1 at both Katanning (*P* = 0.042) and Merredin (*P* = 0.002) relative to NS (Figure 5a). Whole grain Zn and P concentrations did not differ between the three CE‐1 sibling lines and NS at both Merredin and Katanning. All lines (CE‐1 and NS) had higher whole grain Zn and P concentrations at Katanning relative to Merredin, possibly due to lower TGW at Katanning (Figure 5b,c, Table S3). Analysis of NA and DMA showed that whole grain NA concentrations were significantly higher for CE‐1.1 and CE‐1.2 at Katanning (*P* = 0.005 and *P* ≤ 0.001, respectively), and for all three CE‐1 sibling lines at Merredin (*P* = 0.004, *P* = 0.008 and *P* = 0.016, respectively), relative to NS. Whole grain DMA concentrations were significantly higher for CE‐1.1 and CE‐1.2 at Katanning (*P* = 0.014 and *P* = 0.045, respectively), and CE‐1.2 and CE‐1.3 at Merredin (*P* = 0.022 and P ≤ 0.001, respectively), relative to NS (Figure 5d,e).

**Figure 6:**
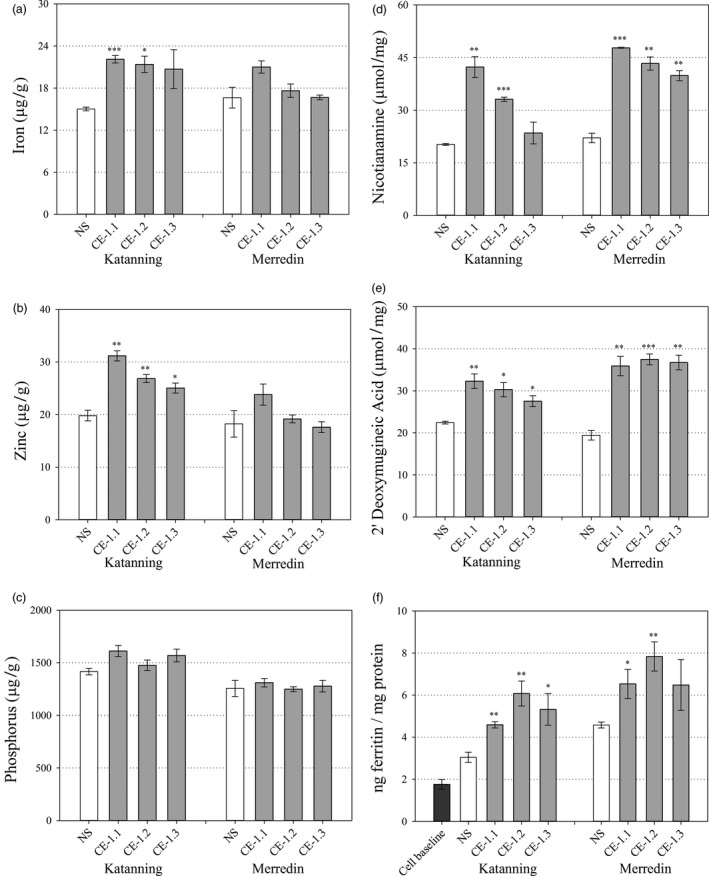
White flour nutrition of field grown CE‐1 and NS wheat lines. Nutrient and metabolite concentrations in white flour samples of NS (white) and three CE‐1 sibling lines (CE‐1.1, 1.2 and 1.3, grey) at the T_6_ generation. (a–c) White flour Fe, Zn and P concentrations (μg/g) of NS and CE‐1 plants grown at Katanning and Merredin field sites. (d, e) White flour NA and DMA concentrations (μmol/mg) of NS and CE‐1 plants grown at Katanning and Merredin field sites. (f) White flour Fe bioavailability of NS and CE‐1 plants grown at Merredin and Katanning field sites. Bars represent mean ± SEM of 3 biological replicates. Asterisks denote the significance between NS and each CE‐1 wheat line for *P* < 0.05 (*), *P* ≤ 0.01 (**), *P* ≤ 0.001 (***) as determined by student's *t*‐test.

White flour Fe concentrations were significantly higher for CE‐1.1 and CE‐1.2 at Katanning (*P* ≤ 0.001 and *P* = 0.012, respectively) relative to NS (Figure 6a) but did not differ between any line at Merredin. White flour Zn concentrations were significantly higher for all three CE‐1 sibling lines at Katanning (*P* = 0.002, *P* = 0.010 and *P* = 0.036, respectively) relative to NS (Figure 6b) but did not differ between any line at Merredin. White flour P concentration did not differ between any line at both Merredin and Katanning (Figure 6a–c). White flour NA concentrations were significantly higher for CE‐1.1 and CE‐1.2 at Katanning (*P* = 0.004 and P ≤ 0.001, respectively) and for all three CE‐1 sibling lines at Merredin (P ≤ 0.001, *P* = 0.002 and *P* = 0.002, respectively), relative to NS (Figure 6d). White flour DMA concentrations were significantly higher for all three CE‐1 sibling lines at both Katanning (*P* = 0.010, *P* = 0.020 and *P* = 0.035, respectively) and Merredin (*P* = 0.006, *P* ≤ 0.001 and *P* = 0.002, respectively) relative to NS (Figure 6e).

### Iron bioavailability is increased in white flour containing increased concentrations of NA and DMA

Iron bioavailability in CE‐1.1, CE‐1.2, CE‐1.3 and NS whole grain and white flour produced from field‐grown grain was determined through ferritin assays of *in vitro* Caco‐2 cells incubated with flour digests. The levels of bioavailable Fe in CE‐1.1, CE‐1.2, CE‐1.3 and NS whole grain flour were negligible and did not differ significantly; a result likely due to phytic acid and other inhibitory compounds in the outer layers of wheat grain (Figure 5f). By contrast, levels of bioavailable Fe were significantly increased in CE‐1.1, CE‐1.2, CE 1.3 white flour digests from Katanning (*P* = 0.005, *P* = 0.009 and *P* = 0.045, respectively), and CE‐1.1 and CE‐1.2 white flour digests from Merredin (*P* = 0.050 and *P* = 0.010, respectively) relative to NS (Figure 6f). White flour Fe bioavailability was significantly correlated with NA concentration (*r* = 0.711, *P* = 0.048) and DMA concentration (*r* = 0.812, *P* = 0.014). White flour Fe bioavailability was not significantly correlated with concentrations of Fe (*r* = 0.242, *P* = 0.563), Zn (*r* = −0.132, *P* = 0.755) and P (*r* = −0.433, *P* = 0.284) (Table 1).

**Table 1:** Pearson's correlation analysis of NA, DMA, Fe, Zn and P concentrations in NS and CE‐1 white flour and ferritin production in Caco‐2 cells

	DMA (μmol/mg)	Fe (μg/g)	Zn (μg/g)	P (μg/g)	Ferritin (ng/mg protein)
NA (μmol/mg)	**0.912** ** 0.002	**0.440** 0.276	**0.239** 0.569	−**0.180** 0.670	**0.711** * 0.048
DMA (μmol/mg)		**0.355** 0.389	**0.132** 0.755	−**0.145** 0.732	**0.812** * 0.014
Fe (μg/g)			**0.893** ** 0.003	**0.627** 0.096	**0.242** 0.563
Zn (μg/g)				**0.854** ** 0.007	−**0.132** 0.755
P (μg/g)					−**0.433** 0.284

Bold and non‐bold numbers represent correlation coefficients and P values, respectively. Asterisks denote significant correlation between factors for *P < 0.05, **P ≤ 0.01, ***P ≤ 0.001.

## Discussion

S‐adenosylmethionine (SAM) is an ubiquitous cosubstrate involved in the production of polyamines, ethylene, biotin, NA and DMA in plants (Mao *et al*., 2015; Roeder *et al*., 2009). Because increased *NAS* gene activity diverts more endogenous SAM towards NA and DMA production, it is important to carefully phenotype Fe and Zn biofortified plants with constitutive NAS expression to identify any pleiotropic effects that may negatively impact on plant phenotype. In our study we analyzed multiple CE‐*OsNAS2* transformation events in several glasshouse experiments, and the lead CE‐1 event in multi‐location field trials in Western Australia, and found that important agronomical traits such as shoot area, plant height, total grain number and TGW did not differ from NS in most cases (Figure 1, Tables S2 and S3). These results are similar to those reported for glasshouse and field‐grown 35S::*OsNAS2* rice (Johnson *et al*., 2011; Trijatmiko *et al*., 2016), as well as glasshouse grown UBI‐1::*OsNAS2* wheat (Singh *et al*., 2017), and demonstrate that constitutive expression of the *OsNAS2* gene to biofortify bread wheat with Fe and Zn does not negatively impact on plant phenotype and yield. Determining whether the changes observed in Fe and Zn accumulation patterns during grain filling (Figure 2) relate to increased remobilization of Fe and Zn from vegetative to grain tissues in CE‐*OsNAS2* plants will require further analysis using radioactive or stable Fe and Zn isotopes (Pottier *et al*., 2014).

The most striking result from our synchrotron X‐ray fluorescence microscopy (XFM) analyses was the increased Fe signal in endosperm tissue of CE‐1 grain relative to NS (Figures 3d and 4d). Line scans showed that the increased Fe signal extended throughout CE‐1 endosperm and did not co‐localize with P (Figures 3f and 4f), indicating that Fe is not associated with phytic acid in this tissue nor the inhibitory effects that phytic acid has on mineral absorption in humans (Hurrell, 2003). By contrast, both CE‐1 and NS grain showed high Zn signal co‐localized with P in aleurone cells and essentially no Zn signal in the endosperm (Figure 3f and 4f). The observed differences in grain Fe and Zn localization demonstrate that wheat white flour contains Zn that is derived almost entirely from outside of the endosperm. We postulate that the wheat crease, which is difficult to remove through milling (Campbell, 2007), represents the major source of Zn in white flour. Line scans across the crease region showed similar Zn signal intensities in CE‐1 and NS modified aleurone cells yet higher Zn signal intensity in the CE‐1 nucellar projection (Figure 3e–f; Figure S4), a tissue which has been shown to contain NA‐bound Fe (De Brier *et al*., 2016), and this difference likely accounts for the observed Zn concentration increase in CE‐1 white flour (Figure 6b). Interestingly, synchrotron XFM studies of rice grain reveal a radically different Zn distribution pattern that extends throughout the endosperm and shows no obvious barrier to endosperm loading (Johnson *et al*., 2011; Kyriacou *et al*., 2014). Determining whether differences in transport mechanisms or sink strength account for the Zn distribution differences between wheat and rice grain merits further investigation.

While flour milled from CE‐1 grain contained significantly higher concentrations of NA and DMA at both Merredin and Katanning field sites, relative to NS, while white flour Fe and Zn concentrations only differed from NS at Katanning (Figure 6a–e). The NS and CE‐1 wheat plants were larger and higher yielding at the Merredin field site (Table S3), which may have had a dilution effect on grain micronutrient concentrations and thereby minimized differences between the genotypes. This result demonstrates the importance of multi‐location field trials for accurate assessment of wheat Fe and Zn biofortification traits, yet also uniquely enabled us to examine the effects of NA and DMA as promoters of Fe bioavailability (Eagling *et al*., 2014b; Glahn *et al*., 1998; Lee *et al*., 2012; Tako *et al*., 2016) under differing Fe concentrations. We used the *in vitro* Caco‐2 cell line model to measure Fe bioavailability in NS and CE‐1 white flour produced at both field sites and found that white flour NA and DMA concentrations were significantly and positively correlated with Fe bioavailability at both sites while white flour Fe concentration was not significantly correlated with Fe bioavailability (Figure 6, Table 1). This result was most evident at the Merredin field trial, where white flour Fe concentrations of two CE‐1 sibling lines (CE‐1.2 and CE‐1.3) were virtually identical to that of NS, however, CE‐1.2 white flour contained 1.7‐fold more bioavailable Fe than NS (Figure 6a and f). In addition to Fe, white flour P concentrations were unchanged between NS and CE‐1 sibling lines, indicating that altered white flour phytate/Fe ratios are not likely to be responsible for the increase in Fe bioavailability (Figure 6c). These results reinforce the finding that NA and/or DMA bind Fe in a readily bioavailable form within wheat white flour (Eagling *et al*., 2014a) and suggests that NA and/or DMA mediate increased uptake of Fe into human cells independent of endosperm Fe concentration. As such, NA and DMA can be viewed as important phytonutrients that increase Fe bioavailability in plant foods (Martin and Li, 2017; Nozoye, 2018), and metabolic engineering strategies focused on increasing their concentrations could help offset the effects of decreased mineral concentrations forecast for C_3_ grains by mid‐century.

## Methods

### Vector construction and generation of wheat transformation events

The full‐length coding sequence of *OsNAS2* (LOC_Os03g19420) was PCR amplified from rice (*Oryza sativa* L.) cv. Nipponbare genomic DNA (Johnson *et al*., 2011). Recombination into a modified pMDC32 vector (Curtis and Grossniklaus, 2003) with the hygromycin phosphotransferase plant‐selectable marker gene placed *OsNAS2* under transcriptional control of the maize (*Zea mays* L.) ubiquitin 1 promoter. Particle bombardment of the construct into immature wheat (*Triticum aestivum* L.) cv. Bobwhite embryos (1.0–1.5 mm in length) was performed at the University of Adelaide (Adelaide, Australia) using established protocols (Kovalchuk *et al*., 2009). Plants were grown in glasshouse conditions (12 h photoperiod, 23 °C day/12 °C night, 50% humidity) in soil (coconut peat and sand mixture) with complete fertilizers.

### Insert copy number analysis

Genomic DNA (10 μg) was isolated from CE‐*OsNAS2* leaf tissue and digested with *Dra*I and *Hind*III restriction enzymes. Restriction fragments were separated by gel electrophoresis (0.8% agarose) alongside a positive barley control and blotted to a nylon membrane. Two independent hybridizations of a ^32^P‐labelled probe to both nopaline synthase terminator and dual 35S promoter were performed using established protocols (Pallotta *et al*., 2014).

### Automated phenotyping

Grain were sown in white plastic pots (14 × 19 cm) containing 2.5 kg of soil mixture (equal parts clay‐loam soil and coconut peat) and Osmocote^®^ fertilizer. Plants were maintained under glasshouse conditions (12 h photoperiod, 24 °C day/18 °C night, 50%–90% humidity) in the phenotyping platform of The Plant Accelerator (Adelaide, Australia). Projected shoot area and plant height were measured 100 days after sowing using the conveyer automated imaging system (Berger *et al*., 2012). Grain number and TGW were manually determined at harvest.

### Inductively coupled plasma optical emission spectrometry (ICP‐OES)

Plant tissues were submerged in 0.1% Tween 20 solution, rinsed with dH_2_O and oven dried for 48 h at 60°C before grinding to a powder using an IKA tube mill (www.ika.com). Inductively coupled plasma optical emission spectrometry (ICP‐OES) analysis was conducted at Waite Analytical Services (Adelaide, SA, Australia), the Robert W. Holley Centre for Agriculture and Health (USDA‐ARS, Ithaca, NY) and the CSBP Soil and Plant Analysis Laboratory (Perth, WA, Australia).

### Quantitative reverse transcription PCR (qRT‐PCR)

Shoot and root tissues (without the crown) of 4‐week‐old plants were separated, cleaned with dH_2_O and snap frozen. Three plants of each genotype (representing one biological replicate) were combined and total RNA was extracted from pulverized frozen plant tissue (100–150 mg) using TRIzol Reagent (Life Technologies, Carlsbad, CA) and a commercial kit (Direct‐zol™; ZymoResearch, Irvine, CA). Genomic DNA was removed from RNA (2 μg) using a DNAse I treatment (Promega, Madison, WI) and reverse transcription was performed using a commercial kit (Bioline).

Consensus primers were designed to amplify homeologous groups of *TaNAS*,* TaNAAT* and *TaDMAS* gene families using Primer3 (http://bioinfo.ut.ee/primer3-0.4.0 ) software. Each biological replicate was analyzed in triplicate and transcripts were quantified against four replicates of 10‐fold serial dilutions (10^2^–10^8^) for each purified PCR template (DNA Clean & Concentrator™‐5; ZymoResearch). Expression levels of *OsNAS2*,* TaNAS*,* TaNAAT* and *TaDMAS* were measured in root and shoot tissues using qRT‐PCR analysis (CFX384‐ BioRad). The geometric mean expression of three housekeeping genes: *TaCyclophilin*,* TaGAPDH* and *TaELF* and *TaGAPDH*,* TaActin* and *TaELF*, was used to normalize *OsNAS2*,* TaNAS*,* TaNAAT* and *TaDMAS* gene expression within shoot and root tissues, respectively (Schreiber *et al*., 2009; Vandesompele *et al*., 2002). All primers had annealing temperatures between 61–65 °C and primer sequences and efficiencies are provided (Table S4).

### Quantification of NA and DMA

Quantification of 6‐aminoquinolyl‐N‐hydroxysuccinimidyl carbamate (AQC) derivatized NA in whole grain was performed via liquid chromatography‐mass spectrometry (LC‐MS) using established protocols (Callahan *et al*., 2007; Johnson *et al*., 2011). Quantification of 9‐fluorenylmethoxycarboxyl chloride (FMOC‐Cl) derivatized NA and DMA in whole grain (Figure 5d,e) and white flour (Figure 6d,e) was performed via RP LC‐MS on an 1290 Infinity II and 6490 Triple Quadrupole LC/MS system (Agilent Technologies Inc., Santa Clara, CA) using established protocols (Selby‐Pham *et al*., 2017). Briefly, sequential methanol (100%) and deionized H_2_O (18MΩ) extractions of pulverized wheat grain or white flour (25 mg) were combined and added (5 μL) to sodium borate buffer (pH = 8, 1 m, 10 μL), EDTA buffer (pH = 8, 50 mm, 10 μL) and fresh FMOC‐Cl solutions (50 mm, 40 μL). After incubation (60 °C, 700 rpm, 15 min), the derivatization reaction was quenched via the addition of formic acid (FA; pH = 4, 5%, 8.9 μL). A Zorbax Eclipse XDB‐C18 Rapid Resolution HS 2.1x100 mm, 1.8 μm particle size column (Agilent Technologies Inc.) was used during chromatography with aqueous (0.1% v/v FA in dH_2_O) and organic (0.1% v/v FA in acetonitrile) mobile phases. For quantification, a stock aqueous solution of NA and DMA (Toronto Research Chemicals, Toronto, ON, Canada) was prepared at 750 μm and a calibration set was generated in the range of 0.005 to 75 μm.

### Analysis of Fe and Zn accumulation post anthesis

Plants were grown in glasshouse conditions (12 h photoperiod, 18 °C day/13 °C night, 40%–80% humidity) in Hortico^®^ potting mix with Osmocote^®^ fertilizer at The University of Melbourne (Victoria, Australia). The main stem flag leaf, rachis, bracts and grain were harvested at 5–8 DAA (days after anthesis), 12–15 DAA, 19–21 DAA, 26–29 DAA and maturity. Samples were washed, oven dried for 48 h at 60°C and ground to a powder before analysis by inductively coupled plasma mass spectrometry (ICP‐MS) at the Environmental Analysis Laboratory (Lismore, NSW, Australia).

### Synchrotron X‐ray fluorescence microscopy (XFM)

Elemental X‐ray fluorescence (XRF) maps of Fe, Zn, Cu and Mn in transverse cross‐sections of two representative CE‐1 and NS grain (four sets of maps total) were collected at the XFM beamline at the Australian Synchrotron (Melbourne, Australia) as previously described (Van Malderen *et al*., 2017). Briefly, the beam energy was set at 15.6 keV and the beam focused to approximately 2 × μm^2^ using Kirkpatrick‐Baez mirrors. Samples were analyzed continuously in the horizontal direction with a sampling interval of 4 μm and a step size of 4 μm in the vertical direction (pixel transit time was set at 5.2 ms). The XRF signal from the 80 μm transverse grain sections was collected using a 384‐element Maia detector system. Tri‐colour elemental maps showing the distribution of Fe, Zn and P near the grain edge of one representative CE‐1 and NS grain (two sets of maps total; different grain from those used with the Maia detector) were collected using a separate Vortex‐EM detector. The tri‐colour maps were used as guides to select rectangular areas of approximately 14 × 140 μm near the grain edge for the generation of Fe, Zn, P and S line scans. Elemental maps were generated using GeoPIXE (http://nmp.csiro.au/GeoPIXE.html) software. The NS grain contained 38 μg/g DW Fe, 71 μg/g DW Zn, 4700 μg/g DW P and 1630 μg/g DW S while CE‐1 grain contained 69 μg/g DW Fe, 122 μg/g DW Zn, 5300 μg/g DW P and 2100 μg/g DW S (Table S5).

### Confined field trials

Confined field trials were conducted in Western Australia from June to December 2015 at the New Genes for New Environment facilities located in Merredin (31.4837° S, 118.2771° E) and Katanning (33.6894° S, 117.5551° E). Grain were sown in 2 m^2^ plots with three replicate plots per genotype and arranged in a randomized block design at each site. Rows were spaced at 30 cm and grain were sown at a rate of 60 kg/ha. At maturity, average plant height was determined from three representative measurements per plot and spike number, total biomass and TGW were determined from 0.15 m^2^ subsamples per plot (Table S3). Grain yield was calculated from the amount of grain harvested per 2 m^2^ plot and extrapolated to kg/ha. Soil properties of both field sites are provided in Table S6.

### Production of white flour

Whole grain samples harvested at Merredin and Katanning were conditioned to 13% moisture content for 24 h prior to milling. Each sample was milled using a Quadrumat Junior laboratory mill (Brabender, Duisburg, Germany) at constant temperature and run through a 280 μm sieve to isolate the white flour fraction. Average flour extraction for all lines from Merredin and Katanning was 71.5 ± 0.2%.

### Caco‐2 cell culture assessment of Fe bioavailability

Whole grain and white flour samples were digested for Caco‐2 cell Fe‐bioavailability analysis as previously described (Glahn *et al*., 1998; Trijatmiko *et al*., 2016). The Caco‐2 cells were maintained in supplemented Dulbecco's modified Eagle medium (DMEM) for 11 days post‐seeding and replaced with supplemented minimum essential media (MEM) solution 48 h prior to the experiment. On the experiment day, gastric‐digested samples (1.5 mL) were added to cylindrical Transwell inserts (Corning Life Sciences, Corning, NY) fitted with a semipermeable (15 000 Da MWCO) basal membrane (Spectra/Por 2.1, Spectrum Medical, Gardena, CA). The inserts were placed within wells containing Caco‐2 cell monolayers and incubated for 2 h (37 °C), after which the inserts were removed and additional MEM (1 mL) added to the cells before incubation for 22 h (37 °C). After incubation, growth medium was removed by aspiration and the Caco‐2 cells were washed twice with a solution (pH = 7.0) containing NaCl (140 mmol/L), KCl (5 mmol/L) and PIPES (10 mmol/L) and harvested with the addition of dH_2_O (1.5 mL) and brief sonication (Lab‐Line Instruments, Melrose Park, IL). In an aliquot of the Caco‐2 cell solution, ferritin content was determined using an immunoradiometric assay (FER‐IRON II Ferritin Assay, Ramco Laboratories, Houston, TX) and total protein content was determined using a colorimetric assay (Bio‐Rad DC Protein Assay, Bio‐Rad, Hercules, CA). As Caco‐2 cells synthesize ferritin in response to intracellular Fe, we used the ratio of ferritin/total protein (expressed as ng ferritin/mg protein) as an index of cellular Fe uptake.

### Statistical analysis

All graphs and statistical analyzes were generated using Minitab^®^ 17 Statistical Software, Minitab, State College, PA and SigmaPlot v13, Systat Software Inc., San Jose. Data are presented as mean ± SEM with biological replicate numbers noted in table and figure legends. Student's *t*‐test was used to determine significant differences between means. Data are available upon request to the corresponding author of this paper.

## Conflict of interest

The authors declare no conflict of interest.

## Supporting information


**Figure S1** Flowchart detailing the analyses performed for each generation of the Ubi::*OsNAS2* lead event (CE‐1) from transformation T_0_ to T_6_ generation.
**Figure S2** Relative quantification of DMA biosynthetic gene transcript levels in NS and CE‐1 shoots (left) and roots (right).
**Figure S3** Concentrations of Fe, Zn, NA and DMA in NS and CE‐1 seedling shoot tissue.
**Figure S4** Tri‐colour elemental maps of Fe, Zn and Cu in transverse cross‐sections of one representative NS and CE‐1 grain.
**Figure S5** Elemental maps of Cu and Mn in transverse cross‐sections of two representative NS and CE‐1 grain.
**Table S1** Iron and zinc concentrations (μg/g DW) in NS and CE‐1 plant tissues at 5–8, 12–15, 19–21 and 26–29 days after anthesis (DAA) as well as maturity.
**Table S2** Biomass (mg DW) of NS and CE‐1 plant tissues at 5–8, 12–15, 19–21 and 26–29 days after anthesis (DAA) as well as maturity.
**Table S3** Agronomic performance of NS and CE‐1 sibling lines grown at the Katanning and Merredin field sites.
**Table S4** Wheat genes and primers used for quantitative reverse transcription PCR (qRT‐PCR) analysis of CE‐1 and NS seedling shoot and root tissue.
**Table S5** Elemental concentrations (μg/g DW) of T_3_ grain harvested from 10 NS and 9 CE‐1 plants including the two batches used for synchrotron XFM analysis (in bold).
**Table S6** Soil properties of Katanning (K) and Merredin (M) field sites.

## References

[pbi13074-bib-0001] Beal, T. , Massiot, E. , Arsenault, J.E. , Smith, M.R. and Hijmans, R.J. (2017) Global trends in dietary micronutrient supplies and estimated prevalence of inadequate intakes. PLoS One, 12, e0175554. 28399168 10.1371/journal.pone.0175554 PMC5388500

[pbi13074-bib-0002] Berger, B. , De Regt, B. and Tester, M. (2012) High‐throughput phenotyping in plants. Methods Mol. Biol. 918, 9–20. 22893282 10.1007/978-1-61779-995-2_2

[pbi13074-bib-0003] Borrill, P. , Connorton, J.M. , Balk, J. , Miller, A.J. , Sanders, D. and Uauy, C. (2014) Biofortification of wheat grain with iron and zinc: integrating novel genomic resources and knowledge from model crops. Front Plant Sci. 5, 1–8. 10.3389/fpls.2014.00053 PMC3930855 24600464

[pbi13074-bib-0004] Bouis, H.E. , Hotz, C. , McClafferty, B. , Meenakshi, J.V. and Pfeiffer, W.H. (2011) Biofortification: a new tool to reduce micronutrient malnutrition. Food Nutr. Bull. 32, S31–S40. 21717916 10.1177/15648265110321S105

[pbi13074-bib-0005] Brouns, F. , Hemery, Y. , Price, R. and Anson, N.M. (2012) Wheat aleurone: separation, composition, health aspects, and potential food use. Crit. Rev. Food Sci. Nutr. 52, 553–568. 22452734 10.1080/10408398.2011.589540

[pbi13074-bib-0006] Callahan, D.L. , Kolev, S.D. , O'Hair, R.A.J. , Salt, D.E. and Baker, A.J.M. (2007) Relationships of nicotianamine and other amino acids with nickel, zinc and iron in *Thlaspi* hyperaccumulators. New Phytol. 176, 836–848. 17897323 10.1111/j.1469-8137.2007.02216.x

[pbi13074-bib-0007] Campbell, G.M. (2007) Chapter 7 Roller milling of wheat. Handb. Powder Technol. 12, 383–419.

[pbi13074-bib-0008] Clemens, S. , Deinlein, U. , Ahmadi, H. , Höreth, S. and Uraguchi, S. (2013) Nicotianamine is a major player in plant Zn homeostasis. Biometals, 26, 623–632. 23775667 10.1007/s10534-013-9643-1

[pbi13074-bib-0009] Connorton, J.M. , Jones, E.R. , Rodríguez‐Ramiro, I. , Fairweather‐Tait, S. , Uauy, C. and Balk, J. (2017) Wheat vacuolar iron transporter TaVIT2 transports Fe and Mn and is effective for biofortification. Plant Physiol. 174, 2434–2444. 28684433 10.1104/pp.17.00672 PMC5543970

[pbi13074-bib-0010] Curtis, M.D. and Grossniklaus, U. (2003) A Gateway cloning vector set for high‐throughput functional analysis of genes *in planta* . Plant Physiol. 133, 462–469. 14555774 10.1104/pp.103.027979 PMC523872

[pbi13074-bib-0011] De Brier, N. , Gomand, S.V. , Donner, E. , Paterson, D. , Smolders, E. , Delcour, J.A. and Lombi, E. (2016) Element distribution and iron speciation in mature wheat grains (*Triticum aestivum* L.) using synchrotron X‐ray fluorescence microscopy mapping and X‐ray absorption near‐edge structure (XANES) imaging. Plant Cell Environ. 39, 1835–1847. 27038325 10.1111/pce.12749

[pbi13074-bib-0012] Eagling, T. , Neal, A.L. , McGrath, S.P. , Fairweather‐Tait, S. , Shewry, P.R. and Zhao, F.J. (2014a) Distribution and speciation of iron and zinc in grain of two wheat genotypes. J. Agric. Food Chem. 62, 708–716. 24382168 10.1021/jf403331p

[pbi13074-bib-0013] Eagling, T. , Wawer, A.A. , Shewry, P.R. , Zhao, F. and Fairweather‐tait, S.J. (2014b) Iron bioavailability in two commercial cultivars of wheat: comparison between wholegrain and white flour and the effects of nicotianamine and 2′‐deoxymugineic acid on iron uptake into Caco‐2 cells. J. Agric. Food Chem. 62, 10320–10325. 25275535 10.1021/jf5026295

[pbi13074-bib-0014] Glahn, R.P. , Lee, O.A. , Yeung, A. , Goldman, M.I. and Miller, D.D. (1998) Caco‐2 cell ferritin formation predicts nonradiolabeled food iron availability in an *in vitro* digestion/Caco‐2 cell culture model. J. Nutr. 128, 1555–1561. 9732319 10.1093/jn/128.9.1555

[pbi13074-bib-0015] Hourston, J.E. , Ignatz, M. , Reith, M. , Leubner‐Metzger, G. and Steinbrecher, T. (2017) Biomechanical properties of wheat grains: the implications on milling. J. R. Soc. Interface 14, 20160828. 28100826 10.1098/rsif.2016.0828 PMC5310733

[pbi13074-bib-0016] Hurrell, R.F. (2003) Influence of vegetable protein sources on trace element and mineral bioavailability. J. Nutr. 133, 2973–2977. 10.1093/jn/133.9.2973S 12949395

[pbi13074-bib-0017] Johnson, A.A. , Kyriacou, B. , Callahan, D.L. , Carruthers, L. , Stangoulis, J. , Lombi, E. and Tester, M. (2011) Constitutive overexpression of the *OsNAS* gene family reveals single‐gene strategies for effective iron‐ and zinc‐biofortification of rice endosperm. PLoS One, 6, e24476. 21915334 10.1371/journal.pone.0024476 PMC3167849

[pbi13074-bib-0018] Kassebaum, N.J. , Jasrasaria, R. , Naghavi, M. , Wulf, S.K. , Johns, N. , Lozano, R. , Regan, M. *et al* (2014) A systematic analysis of global anemia burden from 1990 to 2010. Blood J. 123, 615–625. 10.1182/blood-2013-06-508325 PMC3907750 24297872

[pbi13074-bib-0019] Kovalchuk, N. , Smith, J. , Pallotta, M. , Singh, R. , Ismagul, A. , Eliby, S. , Bazanova, N. *et al* (2009) Characterization of the wheat endosperm transfer cell‐specific protein TaPR60. Plant Mol. Biol. 71, 81–98. 19513805 10.1007/s11103-009-9510-1

[pbi13074-bib-0020] Kyriacou, B. , Moore, K.L. , Paterson, D. , de Jonge, M.D. , Howard, D.L. , Stangoulis, J. , Tester, M. *et al* (2014) Localization of iron in rice grain using synchrotron X‐ray fluorescence microscopy and high resolution secondary ion mass spectrometry. J. Cereal Sci. 59, 173–180.

[pbi13074-bib-0021] Lee, S. , Kim, Y.S. , Jeon, U.S. , Kim, Y.K. , Schjoerring, J.K. and An, G. (2012) Activation of rice nicotianamine synthase 2 (*OsNAS2*) enhances iron availability for biofortification. Mol. Cells, 33, 269–275. 22228185 10.1007/s10059-012-2231-3 PMC3887711

[pbi13074-bib-0022] Lopez, A. , Cacoub, P. , Macdougall, I.C. and Peyrin‐Biroulet, L. (2016) Iron deficiency anaemia. Lancet, 387, 907–916. 26314490 10.1016/S0140-6736(15)60865-0

[pbi13074-bib-0023] Maillard, A. , Diquélou, S. , Billard, V. , Laîné, P. , Garnica, M. , Prudent, M. , Garcia‐Mina, J.M. *et al* (2015) Leaf mineral nutrient remobilization during leaf senescence and modulation by nutrient deficiency. Front. Plant Sci. 6, 1–15. 26029223 10.3389/fpls.2015.00317 PMC4429656

[pbi13074-bib-0024] Mao, D. , Yu, F. , Li, J. , Van de Poel, B. , Tan, D. , Li, J. , Liu, Y. *et al* (2015) FERONIA receptor kinase interacts with S‐adenosylmethionine synthetase and suppresses S‐adenosylmethionine production and ethylene biosynthesis in Arabidopsis. Plant, Cell Environ. 38, 2566–2574. 25988356 10.1111/pce.12570

[pbi13074-bib-0025] Martin, C. and Li, J. (2017) Medicine is not health care, food is health care: plant metabolic engineering, diet and human health. New Phytol. 216, 699–719. 28796289 10.1111/nph.14730

[pbi13074-bib-0026] Moore, K.L. , Zhao, F.J. , Gritsch, C.S. , Tosi, P. , Hawkesford, M.J. , McGrath, S.P. , Shewry, P.R. *et al* (2012) Localisation of iron in wheat grain using high resolution secondary ion mass spectrometry. J. Cereal Sci. 55, 183–187.

[pbi13074-bib-0027] Myers, S.S. , Zanobetti, A. , Kloog, I. , Huybers, P. , Leakey, A.D. , Bloom, A.J. , Carlisle, E. *et al* (2014) Increasing CO2 threatens human nutrition. Nature, 510, 139–142. 24805231 10.1038/nature13179 PMC4810679

[pbi13074-bib-0028] Nozoye, T. (2018) The nicotianamine synthase gene is a useful candidate for improving the nutritional qualities and Fe‐deficiency tolerance of various crops. Front Plant Sci. 9, 1–7. 29636757 10.3389/fpls.2018.00340 PMC5881101

[pbi13074-bib-0029] Pallotta, M. , Schnurbusch, T. , Hayes, J. , Hay, A. , Baumann, U. , Paull, J. , Langridge, P. *et al* (2014) Molecular basis of adaptation to high soil boron in wheat landraces and elite cultivars. Nature, 514, 88–91. 25043042 10.1038/nature13538

[pbi13074-bib-0030] Pottier, M. , Masclaux‐Daubresse, C. , Yoshimoto, K. and Thomine, S. (2014) Autophagy as a possible mechanism for micronutrient remobilization from leaves to seeds. Front. Plant Sci. 5, 1–8. 10.3389/fpls.2014.00011 PMC3900762 24478789

[pbi13074-bib-0031] Prentice, A.M. , Mendoza, Y.A. , Pereira, D. , Cerami, C. , Wegmuller, R. , Constable, A. and Spieldenner, J. (2017) Dietary strategies for improving iron status: balancing safety and efficacy. Nutr. Rev. 75, 49–60. 10.1093/nutrit/nuw055 PMC5155616 27974599

[pbi13074-bib-0032] Roeder, S. , Dreschler, K. , Wirtz, M. , Cristescu, S.M. , van Harren, F.J. , Hell, R. and Piechulla, B. (2009) SAM levels, gene expression of SAM synthetase, methionine synthase and ACC oxidase, and ethylene emission from *N. suaveolens* flowers. Plant Mol. Biol. 70, 535–546. 19396585 10.1007/s11103-009-9490-1 PMC2697359

[pbi13074-bib-0033] Schlemmer, U. , Frølich, W. , Prieto, R.M. and Grases, F. (2009) Phytate in foods and significance for humans: food sources, intake, processing, bioavailability, protective role and analysis. Mol. Nutr. Food Res. 53, 330–375. 10.1002/mnfr.200900099 19774556

[pbi13074-bib-0034] Schreiber, A.W. , Sutton, T. , Caldo, R.A. , Kalashyan, E. , Lovell, B. , Mayo, G. , Muehlbauer, G.J. *et al* (2009) Comparative transcriptomics in the Triticeae. BMC Genom. 10, 285. 10.1186/1471-2164-10-285 PMC2717122 19558723

[pbi13074-bib-0035] Selby‐Pham, J. , Lutz, A. , Moreno‐Moyano, L.T. , Boughton, B.A. , Roessner, U. and Johnson, A.A.T. (2017) Diurnal changes in transcript and metabolite levels during the iron deficiency response of rice. Rice, 10, 14. 28429296 10.1186/s12284-017-0152-7 PMC5398970

[pbi13074-bib-0036] Shewry, P.R. (2009) Wheat. J. Exp. Bot. 60, 1537–1553. 19386614 10.1093/jxb/erp058

[pbi13074-bib-0037] Singh, S.P. , Vogel‐Mikuš, K. , Arčon, I. , Vavpetič, P. , Jeromel, L. , Pelicon, P. , Kumar, J. *et al* (2013) Pattern of iron distribution in maternal and filial tissues in wheat grains with contrasting levels of iron. J. Exp. Bot. 64, 3249–3260. 23918965 10.1093/jxb/ert160 PMC3733147

[pbi13074-bib-0038] Singh, S.P. , Keller, B. , Gruissem, W. and Bhullar, N.K. (2017) Rice NICOTIANAMINE SYNTHASE 2 expression improves dietary iron and zinc levels in wheat. Theor. Appl. Genet. 130, 283–292. 27722771 10.1007/s00122-016-2808-x PMC5263203

[pbi13074-bib-0039] Smith, M.R. , Golden, C.D. and Myers, S.S. (2017) Potential rise in iron deficiency due to future anthropogenic carbon dioxide emissions. GeoHealth, 1, 248–257. 10.1002/2016GH000018 PMC7007116 32158990

[pbi13074-bib-0040] Stomph, T.J. , Choi, E.Y. and Stangoulis, J.C.R. (2011) Temporal dynamics in wheat grain zinc distribution: is sink limitation the key? Ann. Bot. 107, 927–937. 21385780 10.1093/aob/mcr040 PMC3080623

[pbi13074-bib-0041] Takahashi, M. , Terada, Y. , Nakai, I. , Nakanishi, H. , Yoshimura, E. , Mori, S. and Nishizawa, N.K. (2003) Role of nicotianamine in the intracellular delivery of metals and plant reproductive development. Plant Cell, 15, 1263–1280. 12782722 10.1105/tpc.010256 PMC156365

[pbi13074-bib-0042] Tako, E. , Bar, H. and Glahn, R.P. (2016) The combined application of the Caco‐2 cell bioassay coupled with *in vivo* (*Gallus gallus*) feeding trial represents an effective approach to predicting Fe bioavailability in humans. Nutrients, 8, 732. 10.3390/nu8110732 PMC5133116 27869705

[pbi13074-bib-0043] Trijatmiko, K.R. , Dueñas, C. , Tsakirpaloglou, N. , Torrizo, L. , Arines, F.M. , Adeva, C. , Balindong, J. *et al* (2016) Biofortified indica rice attains iron and zinc nutrition dietary targets in the field. Sci. Rep. 6, 1–13. 26806528 10.1038/srep19792 PMC4726380

[pbi13074-bib-0044] Van Malderen, S.J.M. , Laforce, B. , Van Acker, T. , Vincze, L. and Vanhaecke, F. (2017) Imaging the 3D trace metal and metalloid distribution in mature wheat and rye grains via laser ablation‐ICP‐mass spectrometry and micro‐X‐ray fluorescence spectrometry. J. Anal. At. Spectrom. 32, 289–298.

[pbi13074-bib-0045] Vandesompele, J. , De Preter, K. , Pattyn, F. , Poppe, B. , Van Roy, N. , De Paepe, A. and Speleman, F. (2002) Accurate normalization of real‐time quantitative RT‐PCR data by geometric averaging of multiple internal control genes. Genome Biol. 3, RESEARCH0034. 12184808 10.1186/gb-2002-3-7-research0034 PMC126239

[pbi13074-bib-0046] Velu, G. , Ortiz‐Monasterio, I. , Cakmak, I. , Hao, Y. and Singh, R.P. (2014) Biofortification strategies to increase grain zinc and iron concentrations in wheat. J. Cereal Sci. 59, 365–372.

[pbi13074-bib-0047] Waters, B.M. , Uauy, C. , Dubcovsky, J. and Grusak, M.A. (2009) Wheat (*Triticum aestivum*) NAM proteins regulate the translocation of iron, zinc, and nitrogen compounds from vegetative tissues to grain. J. Exp. Bot. 60, 4263–4274. 19858116 10.1093/jxb/erp257

[pbi13074-bib-0048] von Wiren, N. , Klair, S. , Bansal, S. , Briat, J.F. , Khodr, H. , Shioiri, T. , Leigh, R.A. *et al* (1999) Nicotianamine chelates both Fe^III^ and Fe^II^. Implications for metal transport in plants. Plant Physiol. 119, 1107–1114. 10069850 10.1104/pp.119.3.1107 PMC32093

[pbi13074-bib-0049] Zhang, Y. , Shi, R. , Rezaul, K.M. , Zhang, F. and Zou, C. (2010) Iron and zinc concentrations in grain and flour of winter wheat as affected by foliar application. J. Agric. Food Chem. 58, 12268–12274. 21073194 10.1021/jf103039k

[pbi13074-bib-0050] Zheng, L. , Cheng, Z. , Ai, C. , Jiang, X. , Bei, X. , Zheng, Y. , Glahn, R.P. *et al* (2010) Nicotianamine, a novel enhancer of rice iron bioavailability to humans. PLoS One, 5, e10190. 20419136 10.1371/journal.pone.0010190 PMC2855712

