# Supplementary material for: Metabolic engineering of bread wheat improves grain iron concentration and bioavailability
Source: Plant Biotechnol J. 2019 Jan 25;17(8):1514–26. doi: 10.1111/pbi.13074 (PMC6662306; doi:10.1111/pbi.13074)
Supplement: Supplementary file 1 — Figure S1 Flowchart detailing the analyses performed for each generation of the Ubi::OsNAS2 lead event (CE‐1) from transformation T0 to T6 generation. Figure S2 Relative quantification of DMA biosynthetic gene transcript levels in NS and CE‐1 shoots (left) and roots (right). Figure S3 Concentrations of Fe, Zn, NA and DMA in NS and CE‐1 seedling shoot tissue. Figure S4 Tri‐colour elemental maps of Fe, Zn and Cu in transverse cross‐sections of one representative NS and CE‐1 grain. Figure S5 Elemental maps of Cu and Mn in transverse cross‐sections of two representative NS and CE‐1 grain. Table S1 Iron and zinc concentrations (μg/g DW) in NS and CE‐1 plant tissues at 5–8, 12–15, 19–21 and 26–29 days after anthesis (DAA) as well as maturity. Table S2 Biomass (mg DW) of NS and CE‐1 plant tissues at 5–8, 12–15, 19–21 and 26–29 days after anthesis (DAA) as well as maturity. Table S3 Agronomic performance of NS and CE‐1 sibling lines grown at the Katanning and Merredin field sites. Table S4 Wheat genes and primers used for quantitative reverse transcription PCR (qRT‐PCR) analysis of CE‐1 and NS seedling shoot and root tissue. Table S5 Elemental concentrations (μg/g DW) of T3 grain harvested from 10 NS and 9 CE‐1 plants including the two batches used for synchrotron XFM analysis (in bold). Table S6 Soil properties of Katanning (K) and Merredin (M) field sites. [file PBI-17-1514-s001.docx]

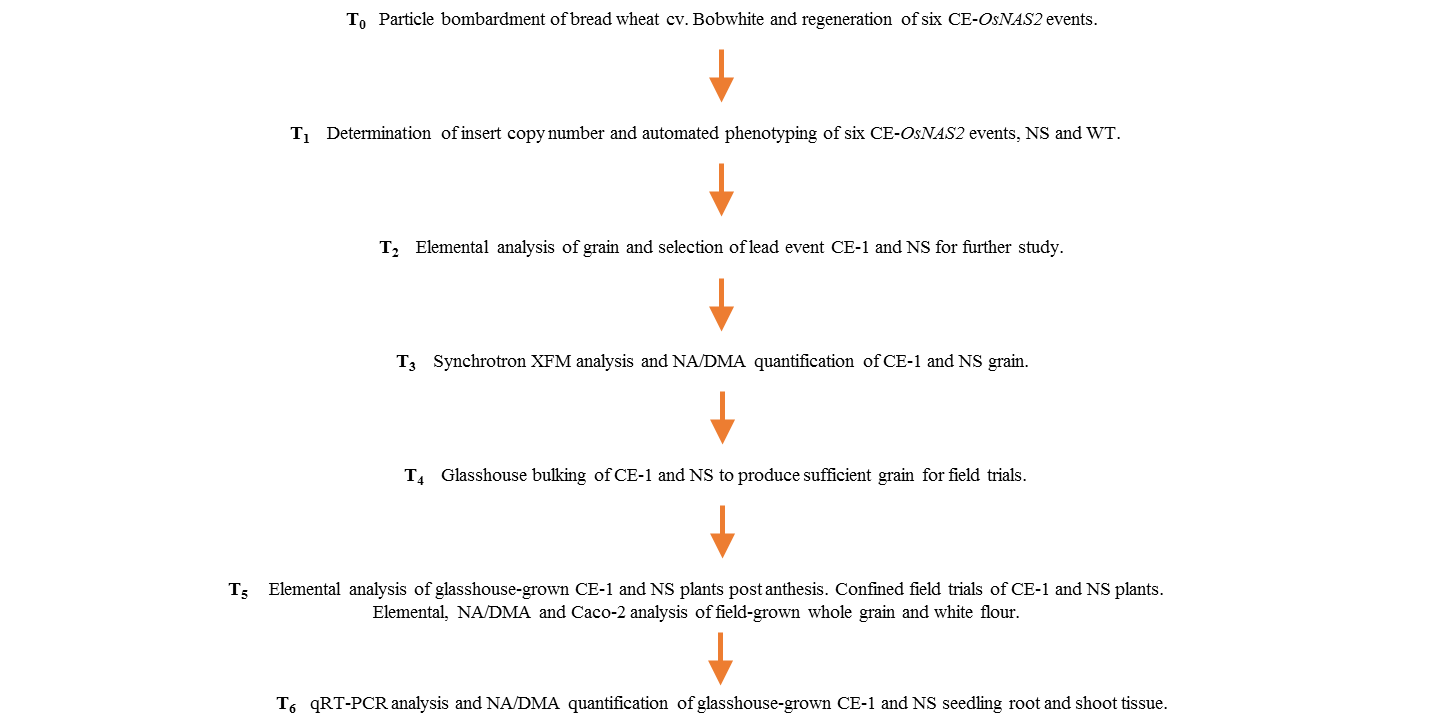


**Figure S1.** Flowchart detailing the analyses performed for each generation of the Ubi::*OsNAS2* lead event (CE-1) from transformation (T_0_) to T_6_ generation. The NS line was derived from CE-1 at the T_1_ generation and included in each subsequent analysis.

**Figure S2.** Relative quantification of DMA biosynthetic gene transcript levels in NS and CE-1 shoots (left) and roots (right). Units on the *y-*axis are mRNA copies per µg of total RNA. Bars represent mean ± SEM of three bulked biological replicates, each with three technical replicates of quantitative RT-PCR.

**Figure S3.** (**a**-**b**) Iron and zinc concentrations (µg g^-1^ DW) in the shoots of NS (white) and CE-1 (orange) plants, respectively. (**c**-**d**) Nicotianamine and 2-deoxymugineic acid concentrations (µmol mg^-1^) in the shoots of NS and CE-1 plants, respectively. Bars represent mean ± SEM of three biological replicates. Asterisks denote the significance between NS and CE-*OsNAS2* events for P < 0.05 (*), P ≤ 0.01 (**), P ≤ 0.001 (***) as determined by student’s t-test.

**Figure S4.** (**a-b**) Bright field images of NS and CE-1 grain sections and corresponding tri-colour XFM elemental maps of Fe (red), Zn (green) and Cu (blue). (**c-d**) Enlarged view of the crease region in NS and CE-1 grain, respectively. The endosperm (E), modified aleurone cells (AL), pigment strand (PS), vascular bundle (VB) and nucellar projection (NP) are labelled.

**Figure S5.** (**a-b**) Bright field images of NS and CE-1 grain sections and corresponding Cu and Mn elemental maps. Colour bar represents high (white) and low (black) elemental concentrations. White boxes in bright field images represent areas used to obtain line scans. (**c**) Line scans showing the distribution and signal intensity of Cu and Mn across NS grain. (**d**) Line scans showing the distribution and signal intensity of Cu and Mn across CE-1 grain. Units for the y-axis are elemental counts per micron.

**Table S1.** Iron and zinc concentrations (µg g^-1^ DW) in NS and CE-1 plant tissues at 5-8, 12-15, 19-21 and 26-29 days after anthesis (DAA) as well as maturity. Values represent mean ± SEM of at least three biological replicates. Asterisks denote the significance between NS and CE-1 for P < 0.05 (*), P ≤ 0.01 (**), P ≤ 0.001 (***) as determined by student’s t-test.

|  |  | **Fe (µg g^-1^)** | | **Zn (µg g^-1^)** | |
| --- | --- | --- | --- | --- | --- |
|  | Timepoint | CE-1 | NS | CE-1 | NS |
| Grain | 5-8 DAA | 40 ± 7.1* | 21 ± 5.5 | 99 ± 5.3* | 83 ± 2.4 |
|  | 12-15 DAA | 52 ± 4.6* | 41 ± 2.2 | 121 ± 11.1* | 91 ± 4.5 |
|  | 19-21 DAA | 30 ± 1.9** | 20 ± 1.3 | 110 ± 4.5*** | 82 ± 3.1 |
|  | 26-29 DAA | 63 ± 4.9 | 51 ± 5.6 | 98 ± 3.5** | 72 ± 5.2 |
|  | Maturity | 71 ± 5.3** | 51 ± 2.5 | 124 ± 7.6** | 90 ± 4.6 |
| Bracts | 5-8 DAA | 17 ± 2.8 | 19 ± 3.4 | 57 ± 4.2* | 47 ± 0.6 |
|  | 12-15 DAA | 29 ± 1.8 | 25 ± 1.4 | 62 ± 3.0*** | 42 ± 2.5 |
|  | 19-21 DAA | 23 ± 7.5 | 11 ± 0.2 | 51 ± 3.0* | 41 ± 2.2 |
|  | 26-29 DAA | 20 ± 1.5 | 17 ± 2.0 | 55 ± 4.3* | 42 ± 1.7 |
|  | Maturity | 21 ± 4.4** | 14 ± 1.2 | 84 ± 11.4 | 76 ± 7.7 |
| Rachis | 5-8 DAA | 118 ± 7.9* | 93 ± 6.9 | 165 ± 11.8* | 127 ± 5.5 |
|  | 12-15 DAA | 42 ± 4.4* | 31 ± 2.9 | 139 ± 15.6* | 93 ± 5.8 |
|  | 19-21 DAA | 68 ± 1.8 | 61 ± 5.4 | 137 ± 10.0** | 101 ± 6.9 |
|  | 26-29 DAA | 17 ± 1.3 | 22 ± 9.5 | 122 ± 17.6* | 81 ± 4.1 |
|  | Maturity | 20 ± 4.7 | - | 73 ± 9.2 | 64 ± 4.1 |
| Flag leaf | 5-8 DAA | 66 ± 4.4 | 79 ± 13.8* | 61 ± 4.6 | 66 ± 2.8 |
|  | 12-15 DAA | 66 ± 1.4 | 63 ± 1.3 | 67 ± 4.7 | 72 ± 8.3 |
|  | 19-21 DAA | 82 ± 11.7 | 62 ± 3.9 | 55 ± 3.1 | 75 ± 4.8** |
|  | 26-29 DAA | 53 ± 2.3 | 50 ± 3.2 | 48 ± 5.3 | 64 ± 1.8** |
|  | Maturity | 28 ± 4.3 | 20 ± 1.1 | 62 ± 6.2 | 49 ± 13.6 |

**Table S2.** Biomass (mg DW) of NS and CE-1 plant tissues at 5-8, 12-15, 19-21 and 26-29 days after anthesis (DAA) as well as maturity. Values represent mean ± SEM of at least three biological replicates. Asterisks denote the significance between NS and CE-1 for P < 0.05 (*), P ≤ 0.01 (**), P ≤ 0.001 (***) as determined by student’s t-test.

|  |  | **Biomass (mg DW)** | |
| --- | --- | --- | --- |
|  | Timepoint | CE-1 | NS |
| Grain | 5-8 DAA | 195 ± 19.4 | 235 ± 32.5 |
|  | 12-15 DAA | 628 ± 40.9 | 764 ± 56.2* |
|  | 19-21 DAA | 1083 ± 68.8 | 1390 ± 100.5* |
|  | 26-29 DAA | 1491 ± 97.1 | 1687 ± 73.7 |
|  | Maturity | 1956 ± 177.1 | 1949 ± 155.3 |
| Bracts | 5-8 DAA | 451 ± 12.6 | 486 ± 44.0 |
|  | 12-15 DAA | 582 ± 25.4 | 537 ± 32.3 |
|  | 19-21 DAA | 574 ± 26.7 | 599 ± 20.6 |
|  | 26-29 DAA | 523 ± 57.8 | 602 ± 63.0 |
|  | Maturity | 627 ± 51.3 | 625 ± 36.4 |
| Rachis | 5-8 DAA | 102 ± 3.0 | 107 ± 6.0 |
|  | 12-15 DAA | 114 ± 7.6 | 117 ± 3.4 |
|  | 19-21 DAA | 128 ± 2.7 | 132 ± 2.8 |
|  | 26-29 DAA | 117 ± 3.4 | 116 ± 3.5 |
|  | Maturity | 120 ± 9.6 | 120 ± 9.8 |
| Flag leaf | 5-8 DAA | 155 ± 8.4 | 175 ± 9.6 |
|  | 12-15 DAA | 187 ± 8.4 | 182 ± 5.9 |
|  | 19-21 DAA | 169 ± 8.9 | 200 ± 12.0* |
|  | 26-29 DAA | 164 ± 9.8 | 175 ± 14.6 |
|  | Maturity | 122 ± 11.6 | 112 ± 15.3 |

**Table S3.** Agronomic performance of NS and CE-1 sibling lines grown at the Katanning and Merredin field sites. Values represent mean ± SEM of three biological replicates. Asterisks denote the significance between NS and CE-1 sibling lines for P < 0.05 (*), P ≤ 0.01 (**), P ≤ 0.001 (***) as determined by student’s t-test.

| **Field Site** | **Genotype** | **Plant height (cm)** | **Spike number (m^2^)** | **Biomass (kg ha^-1^)** | **TGW (g)** | **Grain yield (kg ha^-1^)** |
| --- | --- | --- | --- | --- | --- | --- |
| Katanning | NS | 59.7 ± 2.1 | 320 ± 9 | 5176 ± 488 | 26.1 ± 1.8 | 1358 ± 234 |
|  | CE-1.1 | 51.7 ± 3.6 | 225 ± 31 | 3720 ± 697 | 29.8 ± 1.9 | 860 ± 175 |
|  | CE-1.2 | 56.8 ± 2.9 | 236 ± 22 | 3882 ± 678 | 26.7 ± 2.3 | 846 ± 111 |
|  | CE-1.3 | 58.8 ± 0.8 | 314 ± 9 | 4667 ± 342 | 25.6 ± 1.0 | 1328 ± 195 |
| Merredin | NS | 69.6 ± 0.5 | 419 ± 14 | 10433 ± 870 | 32.7 ± 1.0 | 2633 ± 345 |
|  | CE-1.1 | 65.3 ± 0.3^*^ | 354 ± 22 | 8776 ± 519 | 31.5 ± 0.5 | 1831 ± 343 |
|  | CE-1.2 | 66.7 ± 1.4 | 317 ± 51 | 9176 ± 1236 | 33.0 ± 0.7 | 2120 ± 375 |
|  | CE-1.3 | 70.3 ± 2.0 | 424 ± 5 | 11147 ± 461 | 33.4 ± 2.2 | 2953 ± 698 |

**Table S4.** Wheat genes and primers used for quantitative reverse transcription PCR (qRT‐PCR) analysis of CE-1 and NS seedling shoot and root tissue. The table provides gene name, forward and reverse primer sequences, qRT-PCR primer efficiency and PCR product length.

| **Gene name** | **Forward primer sequence (5' - 3')** | **Reverse primer sequence (5' - 3')** | **Primer efficiency (%)** | **Product length (bp)** |
| --- | --- | --- | --- | --- |
| *TaGAPDH* | TTCAACATCATTCCAAGCAGCA | CGTAACCCAAAATGCCCTTG | 98.1 | 220 |
| *TaELF* | CAGATTGGCAACGGCTACG | CGGACAGCAAAACGACCAAG | 96.3 | 227 |
| *TaWIN* | GGACAGCTTAGGCGAGGAAT | GCTGGGGCTTCCTTAATCTC | 97.7 | 126 |
| *TaVIP2* | AAGGGTGGATGGTGATAGCC | TTGATGTTGCCATGTGCCC | 98.6 | 138 |
| *UbiOsNAS2* | GTTCCAGAAGGCGGAAGAGT | AACGATCGGGGAAATTCG | 95 | 166 |
| *TaNAS1* | GAATGACGTCCGAGGAGAAG | CGATATCGTCCAGCTCCACT | 98.3 | 135 |
| *TaNAS2* | CGGCTTCCTGTACCCCATC | CTCCATCTTGGTGGAGAAGC | 97.7 | 216 |
| *TaNAS3* | TCCAGAAGATCACCGGACTC | CGAGCATGTCGGAGTAGTGC | 96 | 225 |
| *TaNAS4* | GTCTTCCTGGCCGCACTT | GTTCACCACGTCGTCGTCT | 99.6 | 213 |
| *TaNAS5* | GCGGGTTCCTATACCCGAT | TGCATGTCCTTCGACTTGTG | 99.7 | 130 |
| *TaNAS6* | CTCTTCACCGACCTGGTCAC | TGTAGTTGCTGTAGTAGGGGAAGAT | 99.2 | 208 |
| *TaNAS7* | GAGGCGGGTTCGAGGTGCTC | CACCATCTCGCCGAACCT | 92.6 | 179 |
| *TaNAS9* | GAGGAGGCCCTGGTGAAGA | GGATGCAGGACGTCACCA | 99.1 | 118 |
| *TaNAAT1* | CACATTGCCCCTGTCTTGTC | CTGGGTCCGTTGAGACGTTA | 97.3 | 160 |
| *TaNAAT2* | GGACCCAGCAACCTTCATT | GATCCTTCTGGCTTGTGAGG | 96.7 | 165 |
| *TaDMAS* | ATGGAGGAGTGCCACAGG | AGTAGGCGCACAGCTGGAT | 96.1 | 193 |

**Table S5.** Elemental concentrations (µg g^-1^ DW) of T_3_ grain harvested from 10 NS and 9 CE-1 plants including the two batches used for synchrotron XFM analysis (in bold). Asterisks denote the significance between mean values of NS and CE-1 for P < 0.05 (*), P ≤ 0.01 (**), P ≤ 0.001 (***) as determined by student’s t-test.

| Genotype | Plant | Grain number | Al | Ca | Cu | Fe | K | Mg | Mn | Mo | Na | Ni | P | S | Zn |
| --- | --- | --- | --- | --- | --- | --- | --- | --- | --- | --- | --- | --- | --- | --- | --- |
| NS | 1 | 213 | 0.10 | 370 | 7.0 | 44 | 4700 | 1390 | 161 | 1.5 | 7.4 | < 0.4 | 4500 | 1520 | 78 |
|  | 2 | 196 | 0.13 | 430 | 8.3 | 52 | 4900 | 1580 | 176 | 1.5 | 8.2 | < 0.4 | 5100 | 1760 | 91 |
|  | 3 | 318 | 0.19 | 480 | 7.8 | 43 | 4600 | 1510 | 143 | 1.6 | 7.6 | < 0.4 | 4900 | 1580 | 69 |
|  | 4 | 262 | < 0.1 | 450 | 6.3 | 36 | 4600 | 1440 | 168 | 1.6 | 8.0 | < 0.4 | 4600 | 1540 | 64 |
|  | 5 | 243 | 0.14 | 400 | 5.6 | 34 | 4600 | 1310 | 142 | 1.5 | 8.4 | < 0.4 | 4400 | 1340 | 60 |
|  | 6 | 275 | 0.15 | 390 | 6.1 | 35 | 4700 | 1300 | 141 | 1.4 | 7.1 | < 0.4 | 4300 | 1410 | 62 |
|  | 7 | 192 | 0.38 | 450 | 7.3 | 44 | 4400 | 1520 | 179 | 1.5 | 9.7 | < 0.4 | 4700 | 1650 | 79 |
|  | 8 | 211 | 0.16 | 450 | 6.0 | 33 | 4600 | 1390 | 136 | 1.6 | 9.3 | < 0.4 | 4400 | 1450 | 57 |
|  | **9** | **185** | **0.12** | **410** | **6.0** | **38** | **4400** | **1420** | **156** | **1.5** | **8.5** | **< 0.4** | **4700** | **1630** | **71** |
|  | 10 | 240 | < 0.1 | 460 | 8.1 | 39 | 4400 | 1540 | 137 | 1.6 | 8.6 | < 0.4 | 4900 | 1620 | 69 |
|  | Mean | 234 | 0.2 | 429 | 7 | 40 | 4590 | 1440 | 154 | 2 | 8 | na | 4650 | 1550 | 70 |
|  | SEM | 13 | 0.03 | 11 | 0.3 | 2 | 50 | 30 | 5 | 0.02 | 0.25 | na | 82 | 40 | 3 |
| CE-1 | 1 | 246 | 0.33 | 420 | 9.2 | 55 | 4600 | 1620 | 152 | 1.2 | 11 | < 0.4 | 4900 | 1750 | 96 |
|  | 2 | 354 | < 0.1 | 480 | 9.0 | 57 | 4700 | 1530 | 144 | 0.69 | 10 | 0.65 | 4900 | 1730 | 84 |
|  | **3** | **253** | **< 0.1** | **510** | **11** | **69** | **4700** | **1840** | **112** | **0.92** | **9.2** | **0.40** | **5300** | **2100** | **122** |
|  | 4 | 242 | 0.14 | 420 | 7.2 | 38 | 4400 | 1550 | 71 | 1.4 | 11 | < 0.4 | 4600 | 1550 | 76 |
|  | 5 | 254 | < 0.1 | 460 | 8.4 | 44 | 4400 | 1670 | 58 | 1.4 | 9.1 | < 0.4 | 5000 | 1770 | 90 |
|  | 6 | 211 | < 0.1 | 470 | 9.9 | 60 | 4400 | 1770 | 76 | 1.2 | 8.9 | < 0.4 | 5300 | 1970 | 110 |
|  | 7 | 294 | 0.14 | 530 | 11 | 64 | 4600 | 1750 | 107 | 0.83 | 12 | < 0.4 | 5100 | 1990 | 111 |
|  | 8 | 111 | 0.11 | 420 | 16 | 98 | 5900 | 2200 | 131 | 1.3 | 22 | 0.52 | 6000 | 2500 | 192 |
|  | 9 | 276 | < 0.1 | 470 | 9.5 | 64 | 5400 | 1690 | 125 | 0.87 | 13 | < 0.4 | 5400 | 1960 | 114 |
|  | Mean | 249 | 0.18 | 464 | 10*** | 61*** | 4789 | 1736*** | 108** | 1.09*** | 12** | 0.53 | 5167** | 1924*** | 110*** |
|  | SEM | 22 | 0.05 | 13 | 0.78 | 6 | 173 | 67 | 11 | 0.09 | 1 | 0.07 | 133 | 91 | 11 |

^*^elements not listed because below the detection limit: As (<1), Cd (<0.1), Co (<0.4), Cr (<0.2), Pb (<1), Se (<3) and Ti (<0.02).

**Table S6.** Soil properties of Katanning (K) and Merredin (M) field sites. Values represent mean ± SEM of three replicates and are provided in µg g^-1^ DW unless otherwise specified.

| **Field Site** | **Soil Type** | **NH_4+_** | **NO_3-_** | **P** | **K** | **S** | **Cu** | **Fe** | **Mn** | **Zn** | **Al** | **Ca** | **Mg** | **Na** | **B** | **Organic C (%)** | **pH (CaCl_2_)** |
| --- | --- | --- | --- | --- | --- | --- | --- | --- | --- | --- | --- | --- | --- | --- | --- | --- | --- |
| K | Sand | 6.3 ± 0.5 | 9.7 ± 1.9 | 37.7 ± 1.5 | 21.0 ± 2.8 | 4.4 ± 0.4 | 0.7 ± 0.1 | 76.0 ± 1.2 | 6.8 ± 0.4 | 2.0 ± 0.1 | 15.1 ± 1.3 | 62.6 ± 4.8 | 28.8 ± 1.5 | 9.2 ± 0.0 | 0.4 ± 0.0 | 1.5 ± 0.1 | 4.6 ± 0.1 |
| M | Sand | 4.7 ± 1.0 | 1.7 ± 0.3 | 65.0 ± 2.5 | 113.7 ± 11.8 | 3.9 ± 0.1 | 0.7 ± 0.0 | 123.4 ± 3.5 | 3.4 ± 0.1 | 1.8 ± 0.1 | 17.3 ± 1.9 | 87.3 ± 8.2 | 38.4 ± 3.0 | 8.4 ± 1.3 | 0.8 ± 0.0 | 1.1 ± 0.1 | 4.6 ± 0.0 |
